# Beta Electroencephalographic Oscillation Is a Potential GABAergic Biomarker of Chronic Peripheral Neuropathic Pain

**DOI:** 10.3389/fnins.2021.594536

**Published:** 2021-02-26

**Authors:** Micael Teixeira, Christian Mancini, Corentin Aurèle Wicht, Gianluca Maestretti, Thierry Kuntzer, Dario Cazzoli, Michael Mouthon, Jean-Marie Annoni, Joelle Nsimire Chabwine

**Affiliations:** ^1^Neurology Unit, Medicine Section, Laboratory for Cognitive and Neurological Science, Department of Neuroscience and Movement Science, Faculty of Science and Medicine, University of Fribourg, Fribourg, Switzerland; ^2^Faculty of Medicine, University of Bern, Bern, Switzerland; ^3^Department of Orthopedics, Fribourg Hospital, Fribourg, Switzerland; ^4^Nerve-Muscle Unit, Neurology Service, Department of Clinical Neurosciences, Lausanne University Hospital (CHUV), University of Lausanne, Lausanne, Switzerland; ^5^Gerontechnology and Rehabilitation Group, ARTORG Center for Biomedical Engineering Research, University of Bern, Bern, Switzerland; ^6^Perception and Eye Movement Laboratory, Department of Neurology, Inselspital, Bern University Hospital, University of Bern, Bern, Switzerland; ^7^Division of Neurorehabilitation, Fribourg Hospital, Fribourg, Switzerland

**Keywords:** neuropathic pain, chronic pain, EEG, GABA, Beta oscillation, biomarker

## Abstract

This preliminary investigation aimed to assess beta (β) oscillation, a marker of the brain GABAergic signaling, as a potential objective pain marker, hence contributing at the same time to the mechanistic approach of pain management. This case–control observational study measured β electroencephalographic (EEG) oscillation in 12 right-handed adult male with chronic neuropathic pain and 10 matched controls (∼55 years). Participants were submitted to clinical evaluation (pain visual analog scale, Hospital Anxiety, and Depression scale) and a 24-min high-density EEG recording (BIOSEMI). Data were analyzed using the EEGlab toolbox (MATLAB), SPSS, and R. The global power spectrum computed within the low (Lβ, 13–20 Hz) and the high (Hβ, 20–30 Hz) β frequency sub-bands was significantly lower in patients than in controls, and accordingly, Lβ was negatively correlated to the pain visual analog scale (*R* = −0.931, *p* = 0.007), whereas Hβ correlation was at the edge of significance (*R* = −0.805; *p* = 0.053). Patients’ anxiety was correlated to pain intensity (*R* = 0.755; *p* = 0.003). Normalization of the low and high β global power spectrum (GPS) to the GPS of the full frequency range, while confirming the significant Lβ power decrease in chronic neuropathic pain patients, vanished the significance of the Hβ decrease, as well as the correlation between Lβ power and pain intensity. Our results suggest that the GABAergic Lβ EEG oscillation is affected by chronic neuropathic pain. Confirming the Lβ GPS decrease and the correlation with pain intensity in larger studies would open new opportunities for the clinical application of gamma-aminobutyric acid-modifying therapies.

## Introduction

Pain is a major public health challenge, and a large number of available analgesic drugs do not prevent from high treatment failure rate (Mantha et al., 1993). Despite the great variety of pain etiologies, all pain types are treated using similar drugs, irrespective of their underlying mechanisms (Vargas-Schaffer, 2010), which could explain why successful analgesia is hard to reach. A mechanism-based classification of pain could probably help improving treatment efficiency. Several neurotransmitters are involved in nociception (Schaible, 2007; Ossipov et al., 2010), including gamma-aminobutyric acid (GABA), the major brain inhibitory neurotransmitter, which plays a critical role in different pain processing steps (Enna and McCarson, 2006; Barr et al., 2013). Thus, the GABAergic signaling could be a suitable candidate for a mechanism-based approach to characterize and manage pain.

Pain can be evaluated by means of several clinical tools, among which the Visual Analog Scale (VAS), the Numerical Rating Scale (Haefeli and Elfering, 2006; Hawker et al., 2011), and the Brief Pain Inventory (BPI), the latter taking also into account the complexity and the impact of pain on multiple domains of life, such as sleep and mood (Cleeland and Ryan, 1994; Tan et al., 2004; Shi et al., 2017). These scales are all based on the patient’s subjective pain perception, which is influenced by many factors, such as the cognitive state, mood, etc. On the other hand, many brain areas contributing to pain regulation (within the pain matrix) are also involved in several other functions, such as cognition, emotion, motivation, or other sensory modalities (Ossipov et al., 2010). For all these reasons, the pain matrix structure and function (and thereby pain perception) can be influenced by all the factors mentioned earlier (Bushnell et al., 2013). This complex interplay between factors interfering with both pain regulation and its subjective perception makes it difficult to find a comprehensive assessment tool. Hence, the need for a complementary evaluation, based on more objective criteria, e.g., biomarkers related to physiological pain mechanisms, that could contribute to characterize further and hopefully better manage pain (Archibald et al., 2018).

Successful analgesia is achieved when pain intensity (evaluated by the VAS) is <3 (Mantha et al., 1993). This pain level could be considered as a cutoff value defining in a very simple way what a “significant” pain is, even if other classifications of pain severity exist (Mantha et al., 1993; Collins et al., 1997). The International Association for Studies of Pain defines chronic pain as lasting for more than 3 months (Fine, 2011; Treede et al., 2015). However, the concept of chronic pain refers to the prolonged duration of pain because substantial and long-lasting changes are thought to occur within the pain matrix in this context (Schaible, 2007; Baliki et al., 2012; Baliki et al., 2014). Animal experimental data show that, in chronic pain situations, virtually the whole set of neurotransmitters (including GABA and glutamate) modify their functioning in pain regulating regions. This ultimately leads to an imbalance between excitation and inhibition in favor of excitation (Ossipov et al., 2010; Pinheiro et al., 2016). Therefore, one could expect the brain inhibitory input (mainly mediated by the GABAergic signaling) to lower under these conditions. Some studies show indeed a decrease in cortical inhibition correlated to pain intensity (Barr et al., 2013; Henderson et al., 2013; Parker et al., 2016), whereas others show the opposite (i.e., an increase of inhibition) (Sarnthein et al., 2006; Lim et al., 2016). Most of these preliminary studies were conducted in patients suffering from the pain of different origins, using varied electrophysiological and brain imaging techniques and methods, and data analysis was most of the time not standardized.

Brain GABAergic activity can be measured *via* several techniques, including transcranial magnetic stimulation (Premoli et al., 2014), and electroencephalography (EEG), a noninvasive, cheap, and readily accessible tool to assess brain electrical activity (Barr et al., 2013). The beta (β, 13–30 Hz) and gamma (γ, 30–100 Hz) EEG oscillations represent an index of the brain’s GABAergic activity, as they are mainly produced by inhibitory interneurons (Gaetz et al., 2011; Christian et al., 2015; Baumgarten et al., 2016). Electroencephalographic studies of pain-related GABAergic signaling focus mostly on the γ frequency domain, although γ waves are generated deep in the brain and are therefore not easy to record with scalp EEG (Rutkove, 2007). On the contrary, data concerning pain-related β EEG activity are scarce (Barr et al., 2013; Bushnell et al., 2013; Pinheiro et al., 2016), whereas β activity, related to the amount of GABA in the somatosensory cortex (Baumgarten et al., 2016), is more reliably measured by scalp EEG.

Thus, brain GABAergic activity can be reliably measured by EEG β oscillations. In a mechanistic approach of pain assessment, β oscillations could represent an index of the GABAergic component of pain. This pilot case–control study aimed, therefore, at recording β EEG oscillations in patients suffering from chronic neuropathic pain (CNP), with the hypothesis that β waves would decrease and be negatively correlated with pain intensity, in accordance with most convincing data (Harris and Clauw, 2012; Barr et al., 2013; Parker et al., 2016).

## Materials and Methods

### Study Design

This case–control cross-sectional, observational study involved patients suffering from CNP and sex (all males)-matched healthy controls of similar age (±5 years). Participants were recruited and investigated between September 2015 and January 2018.

### Participants

Patients (all right-handed) suffered from CNP due to a peripheral nerve lesion in the lower limbs lasting for ≥6 months. The diagnosis of peripheral neuropathy was made by a specialist (neurologist, pain specialist, or an orthopedist specialized in spine surgery) and supported by a “Douleur Neuropathique 4 questions” (DN4) (Bouhassira et al., 2005; Bennett et al., 2007) score of ≥4. Exclusion criteria were the presence of any central nervous system lesion or disease (including epilepsy, parasomnia, and cognitive impairment), the coexistence of other types of pain, and analgesic surgical interventions within less than 6 months before inclusion. Sex- and overall age-matched controls [independent *t*-test: *t*(16.955) = 0.358; *p* = 0.738] fulfilled the same inclusion (except for the presence of pain) and exclusion criteria as patients.

The study fulfilled the requirements of the Declaration of Helsinki and was approved by the Ethics Committee of Vaud under the reference 331/15. Each participant signed informed consent before any investigation.

### Data Collection

#### Interview, Clinical Examination, and Clinical Scores

In total, 16 CNP patients and 14 healthy controls were screened. Among them, two controls were finally excluded because they happened to have exclusion criteria upon a second check. All recruited participants underwent individual, in-person interview following a standardized questionnaire including personal and medical data and information on their sleep behavior. In addition, sleep-related [Insomnia Severity Index (Omachi, 2011)] and mood-related [Hospital Anxiety and Depression (HAD) (Zigmond and Snaith, 1983)] scales were compiled. Three additional scales related to pain were included in patients’ assessment: the DN4, to confirm the neuropathic pattern of pain (Bouhassira et al., 2005; Bennett et al., 2007), the VAS, to quantify pain intensity (Collins et al., 1997), and the BPI, French version (Poundja et al., 2007), to further characterize pain and evaluate its impact on patients’ life (Kerns et al., 1985). For the purpose of the analysis included in this paper, only data related to pain and mood were considered.

Clinical examination was performed by medical doctors (JNC and JMA) or one trained medical student (MT) to confirm lower-limb localization of pain and absence of exclusion criteria.

#### Electrophysiological Recordings

**FIGURE 1:**
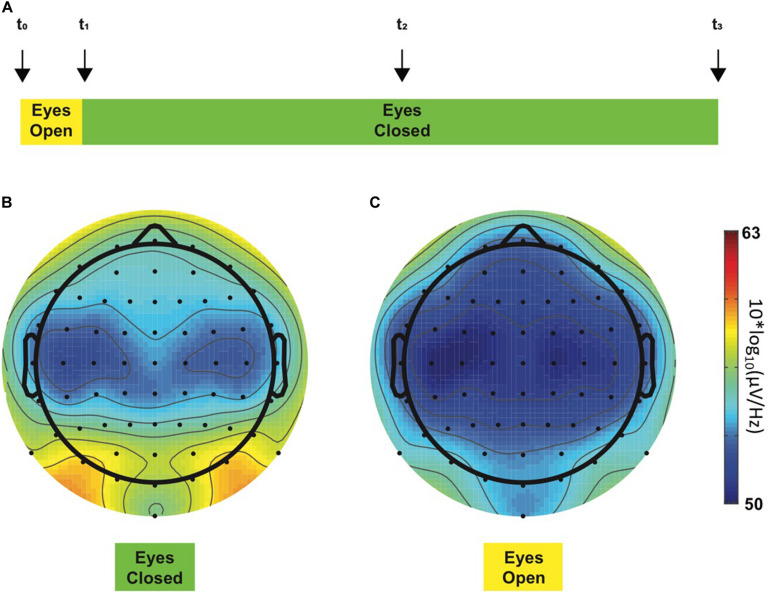
Resting-state EEG recording protocol **(A)**. This 24-min experiment started with a 2-min period, during which the participant opened his eyes (yellow color), and for the rest of the experiment, the eyes had to remain closed (green color). Four acoustic cues (“beeps,” vertical arrows) were presented to the participant during the experimental session to warn him about the ongoing experimental step, i.e., to switch to the eye-closed condition or to keep him awake. Each participant was instructed concerning the meaning of each acoustic cue before the beginning of the experiment. First and last beeps (*t_0* and *t_3*, respectively) determined the beginning and the end of the experimental session, respectively. Second beep (*t_1*) occurred 2 min after *t_0* and instructed the participant to close his eyes, whereas the third beep (*t_2*) occurred in the middle of the experiment and was implemented to maintain the participants awake as they tend to fall asleep. Alpha GPS-derived topoplots in all chronic neuropathic pain patients during eye-closed **(B)** and eye-open **(C)** experimental steps. The color bar at the right side of the figures represents ascending alpha power values from the lowest at the bottom [blue, 50 10*log_10_(μV/Hz)] through the highest at the top [red, 63 10*log_10_(μV/Hz)]. As expected, two alpha peaks were observed in posterior regions on the left and the right side, respectively, during the eyes-closed condition (**B**, left topoplot). On the contrary, these prominent peaks disappeared when the eyes were open (**C**, right topoplot). GPS significantly differed between eye-closed [−0.02 (4.21) dB] and eye-open [−2.17 (4.15) dB] conditions [*t*(11) = −4.072, *p* = 0.002].

All EEG recordings took place in the morning to exclude bias due to nychthemeral variations of pain (Strian et al., 1989). During EEG recordings, participants sat alone in a quiet room shielded in a Faraday cage and were requested to minimize eye blinks or any other body movements. The EEG recording protocol consisted of a 24-min resting-state paradigm (i.e., without any task involvement), mostly with eyes closed (Figure 1A), a condition considered to reflect a basal arousal state and accompanied by an increased alpha frequency amplitude (Barry et al., 2007). The alpha (α) global power spectrum (GPS) averaged over all patients for epochs recorded in eye-closed (Figure 1B) vs. in eye-open (Figure 1C) conditions appeared to be different. The visual difference observed between the two topoplots was indeed statistically confirmed, showing ∼100 times higher power with eyes closed (*p* = 0.002). Similar results were observed in controls (more than 10 times higher GPS in the eye-closed condition; not shown in the figure): 2.07 (3.81) dB for the eyes-closed condition and −0.13 (3.42) dB for the eyes-open condition [*t*(9) = −4.186, *p* = 0.002]. Thus, lower α amplitudes in the eye-open condition confirm significant changes occurring in EEG signals, which could have introduced a bias in our analysis of other frequency bands if not taken into account. For this reason, only data recorded in the eye-closed condition were considered when performing all remaining analyses.

The EEG data were continuously recorded at 1,024 Hz using a high-density (64 electrodes) EEG (BIOSEMI ActiveTwo) recording system referenced to the built-in CMS-DRL ground (BIOSEMI, Amsterdam, Netherlands).

### Data Analysis

### Electroencephalography Preprocessing

#### Spectral Analysis

Automated data preprocessing was performed using a customized MATLAB script based on the EEGLab Toolbox (Delorme and Makeig, 2004) and on the preprocessing pipeline developed by Bigdely-Shamlo et al. (2015) First, raw EEG data were re-referenced to the Cz electrode, down-sampled to 512 Hz, and band-pass filtered between 1 and 60 Hz for artifact removal preparation (Winkler et al., 2015). Automated artifact rejection algorithms (EEGLab plugins) were applied to remove sinusoidal noise stemming from alternating current power line fluctuations [CleanLine (Mullen, 2012)] or high amplitude eyes movements, muscle artifacts, and electrode drifts [Artifact Subspace Reconstruction (Chang et al., 2018)]. Channel interpolation algorithms were used to detect any flawed channels (limited to a maximum of seven rejected electrodes). Preprocessed EEG data were then segmented in 2-s epochs, and two additional artifact detection algorithms were applied to ensure adequate cleaning of epoched data. Finally, re-referencing to average reference was mandatory before all spectral analyses.

It was important to analyze only data recorded in wakefulness because sleep stages were expected to interfere with fast EEG oscillations, including beta waves (Barry et al., 2007). Data analysis was based on a windowed Fourier transform. This was performed with MATLAB and the EEGLab toolbox for MATLAB.

To automatically select data recorded in wakefulness state, a data-driven analysis was developed following empirical evidence (Diaz et al., 2016). This method computes the ratio between the α (8–12 Hz) and the Theta (θ, 5–7 Hz) GPS. For this purpose, a 4-s windowed fast Fourier transform was performed (on 4-s windows obtained by regrouping adjacent 2-s epochs). Only 4-s windows with α/θ ratio ≥1 were considered to be recorded in wakefulness and selected for further analyses. Selected epochs were finally submitted to a 2-s windowed Fourier transformation for frequency analysis between 2 and 48 Hz to minimize electronic devices’ noise and because all frequencies of interest were below 48 Hz.

In the present paper, only data concerning the β (13–30 Hz) and the delta (δ, 2–4 Hz) frequency range are presented. The δ frequency was selected as a negative control, owing to the absence of clear links between δ oscillations and chronic pain in the literature (Pinheiro et al., 2016). The GPS for a given frequency was computed by averaging the respective frequency power data across all epochs recorded over all electrodes in a particular participant. A logarithmic transformation was used to obtain near-normally distributed data (Gasser et al., 1982). Unless otherwise specified, all data are presented in this log-transformed version. Data were then converted to decibel to remove any artefactual constant activity in the signal (represented in the results section as 10^∗^log_10_) (Delorme and Makeig, 2004).

Initial analyses evidenced that the β frequency domain was characterized by two prominent “peaks” (maxima) of power in most subjects, located approximately at 16 and 25 Hz, which corresponded to the centers, respectively, of the Lβ (13–20 Hz) and the Hβ (20–30 Hz) frequency sub-bands (von Rotz et al., 2017). Therefore, all subsequent spectral analyses were performed separately in the two defined β sub-bands.

#### Data Normalization

Normalization of computed Lβ and Hβ GPS to the GPS over the total analyzed frequency range (1–60 Hz; GPS_total_) was performed as follows: first, the logarithmic transformation used in the analysis of the non-normalized data to obtain near-normally distributed data was inverted, after that, the respective ratio between Lβ (GPS_lβ_) and Hβ GPS (GPS_hβ_) and the GPS_total_ were computed, and finally, the logarithmic transformation of the ratio was computed back to obtain the normalized GPS (GPS_normalized_). The following formula summarizes the normalization procedure for the Lβ GPS: Lβ GPS_normalized_ = 10^∗^log_10_ [10^∗∗^(GPS_lB_/10)/10^∗∗^(GPS_total_/10)].

#### Beta Power Maxima and Minima Localization

To localize the power maxima on the scalp (i.e., in the sensor space), the horizontal head section was divided into four quadrants in a way that avoided misleading or ambiguous interpretation (adapted from Grabner and De Smedt, 2012). After averaging β power over all electrodes in each quadrant (following the same procedure as for the GPS; see spectral analysis earlier), the computed GPS was named GPS_q_. The quadrant with the highest power was considered to be the localization of the maximal β GPS_q_ (GPS_qmax_). The procedure to compute the minimal GPS (GPS_qmin_) was exactly the same as for the GPS_qmax_, except that the minimum power was depicted instead of the maximum.

### Statistical Analyses

Statistical analyses were performed using the SPSS 24.0 software (IBM SPSS Statistics). All log-transformed EEG data (GPS) were successfully tested for normality (Kolmogorov–Smirnov test and visual inspection of data distribution). Unless otherwise specified, data are expressed as mean (SD). Differences among δ, Lβ, and Hβ frequency bands within groups were assessed using a multivariate analysis of variance. Group differences were evaluated using *post-hoc* Student’s *t*-test, and the significance threshold (set at *p* < 0.05, 95% confidence interval) was corrected for multiple comparisons with the Bonferroni method (α_*c*_ = 0.017). Correlations were assessed using the Pearson’s *r* correlation coefficient (two-sided).

## Results

### General Data

All participants underwent a formal interview and participated in the EEG assessment. However, only 12 patients and 10 controls were retained for the final analysis [mean age 54.4 (8.0) and 56.3 (15.6) years, respectively], as four patients had a DN4 score <4, whereas two controls suffered from low-intensity pain at the time of EEG assessment. Unless otherwise specified, analyses were performed in all CNP patients (*n* = 12) and/or in all controls (*n* = 10) as appropriate.

Patients had a DN4 score distributed between 4 and 8 [5.7 (1.3)]. Table 1 gives an overview of pain-causing peripheral neuropathy types and etiologies. The great majority of patients suffered from painful radiculopathy related to spine arthrosis, whereas the few remaining cases had polyneuropathy due to diabetes or suspect of paraneoplastic origin. Most patients also reported other sensory symptoms such as tingling, prickling, hypoesthesia, etc. Less than half of them benefited from nonsurgical treatments alone or in combination (nonsteroidal anti-inflammatory drugs, antidepressants, opiates, or even alternative treatment methods). Table 2 shows additional individual information on pain intensity recorded *via* the VAS. The level of pain on the day of the EEG recording (VAS_d_) was in general slightly lower than pain intensity perceived on average during the last 2 weeks [VAS_2w_; respectively, 3.2 (2.1) and 4.2 (1.6)/10; *p* = 0.05 with a paired *t*-test]. Answers to question no. 6 of the BPI questionnaire [French version (Poundja et al., 2007)] corresponded exactly to the VAS_d_ (Tittle et al., 2003) (*R* = 0.992, *p* < 0.001), whereas the level of pain in general (question no. 5 of the BPI questionnaire) was similar to the VAS_2w_ (*R* = 0.982, *p* < 0.001), further confirming that the VAS_2w_ represented the intensity of pain perceived by patients in general.

**TABLE 1:** Patients’ information concerning pain etiologies, associated sensory symptoms, and analgesic treatment.

Clinical picture	Etiologies	Total = 12
Polyneuropathy	Diabetes	1
	Paraneoplastic/toxic	1
	Paraneoplastic/idiopathic	1
	**Subtotal**	3
Lumbar/sacral neuralgia	Spine arthrosis with root compression	9
	**Subtotal**	9
Sensory symptoms	Type of symptoms	n/12
	Tingling	12
	Numbness	9
	Itching	3
	Prickling	10
	Tactile hypoesthesia	6
	Pain hypoesthesia	9
Non-surgical treatment	Type of treatment	n/12
	None	4
	NSAID	3
	Opiates	4
	Antidepressant	3
	Pregabalin	4
	Other drugs	2
	Physical and alternative	3

*NSAID, Nonsteroidal anti-inflammatory drugs.*

**TABLE 2:** Pain intensity scores (significant levels in bold) and predominating pain side in CNP patients.

Patients no.	VAS_d_	VAS_2w_	Pain side
1	1	**3**	R
2	2.5	**3**	R
3	0.5	**5**	L
4	**3**	**5**	R
5	1.5	2	L
9	**8**	**8**	R
11	2.5	**4**	L
12	2.5	**3**	L
13	**3**	**6**	L
14	**3.5**	**4**	R
15	**4**	**3**	L
16	**6.5**	**5**	R
Mean	3.22.1	4.2 1.6	50%R; 50%L

*CNP, chronic neuropathic pain; VAS_d_, Visual Analog Scale assessed the day of the EEG recording; VAS_2w_, VAS referring to the 2 weeks before the EEG recording day; L, left; R, right.*

### Spectral Analysis

**FIGURE 2:**
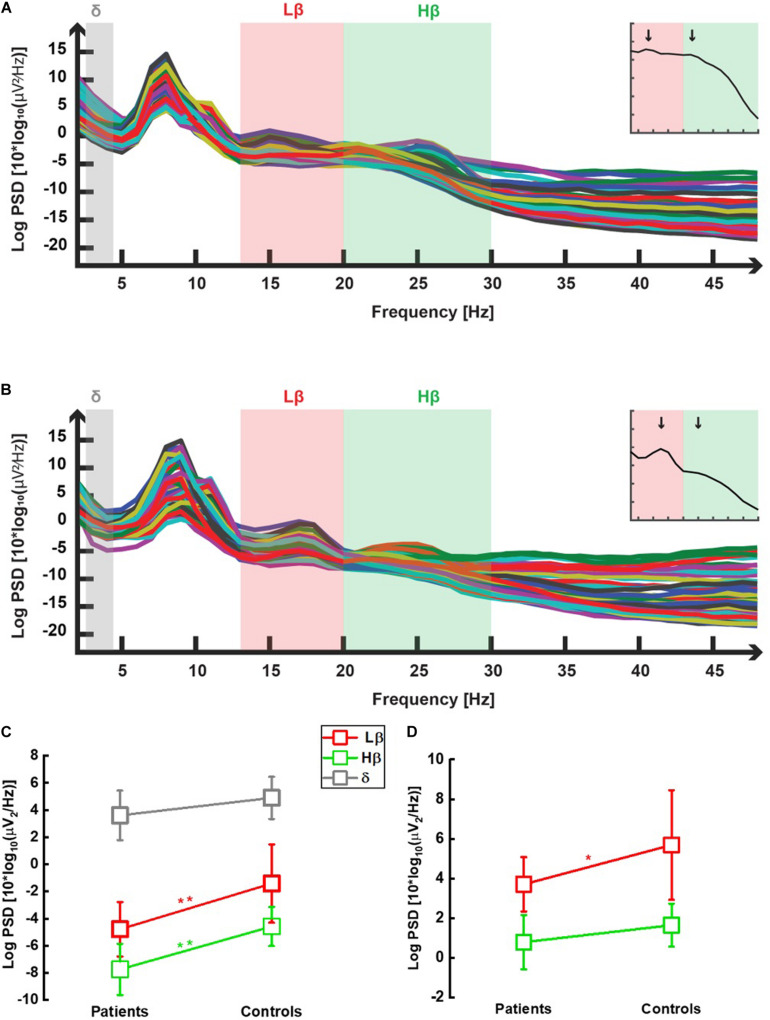
Power spectral densities averaged over all selected epochs in one chronic neuropathic pain patient **(A)** and in one control **(B)**. In both graphs, power spectral densities obtained in the eye-closed condition, after selection of epochs recorded in wakefulness and 2 s-windowed-Fourier transform between 2 and 48 Hz (see section “Materials and Methods”), were plotted against the whole analyzed frequency spectrum. Each trace represents one channel (i.e., one electrode). One prominent peak is observed as expected, in the α range (8–12 Hz) (cf. eye-closed condition). Two additional peaks were seen within the low β (Lβ; 13–20 Hz, light red color) and high β (Hβ; 20–30 Hz, light green color) sub-bands, both in patients **(A)** and in controls (B). No peak was observed in the delta (δ; 2–4 Hz, light gray) frequency range. β peaks occurred, respectively, approximately 16 and 25 Hz in most participants, as illustrated by the two vertical black arrows in the two insets showing the GPS computed over all frequencies derived from each main graph, respectively. GPS in the low β (Lβ), high β (Hβ), and δ frequency ranges for chronic neuropathic pain patients and healthy controls. All data are shown as mean (SD). Non-normalized data **(C)**: Patients displayed lower Lβ (13–20 Hz, red open squares) GPS than controls [−4.7 (2.0) dB vs. 1.4 (2.9) dB] in a highly significant manner [*t*-test: *t*(20) = −3.226, *p* = 0.004]. Likewise, the difference between patients’ Hβ (20–30 Hz, green open squares) GPS [−7.7 (1.9) dB] and controls [−5.4 (1.2) dB] was highly significant [*t*-test: *t*(20) = −3.271, *p* = 0.004]. Within the δ (2–4 Hz, gray open squares) range, no significant GPS difference was observed between patients and controls [2.0 (1.7) and 3.4 (1.7) dB, respectively, *t*(20) = 1.919, *p* = 0.069]. Normalized data **(D)**: a significant decrease in Lβ (13–20 Hz, red open squares) GPS was noticed in patients compared with controls (GPS values are positive due to computation of ratio between two negative numbers): 3.7 (1.4) dB vs. 5.7 (2.8) dB) [*t*-test: *t*(20) = −2.186, *p* = 0.041]. In the Hβ (20–30 Hz, green open squares) range, the decrease was not anymore significant: 0.8 (1.4) vs. 1.6 (1.1) [*t*-test: *t*(20) = −1.610, *p* = 0.123].

In all 22 participants, power spectral densities were computed following windowed fast Fourier transforms after appropriate preprocessing (see section “Materials and Methods”). The output of this analysis is shown in one illustrative patient and one control, respectively, in Figures 2A,B. The frequencies of interest are highlighted in gray (δ), red (Lβ), and green (Hβ) colors. In addition to the prominent α peak (see section “Materials and Methods”), the two peaks defining the Lβ and the Hβ frequency sub-bands (von Rotz et al., 2017) (see also the insets of these two figures representing the GPS over the whole frequency spectrum) are visible around 16 and 25 Hz. Lower power in the Lβ and the Hβ domains in patients compared with controls can already be suspected at this stage (inset graph scales are similar).

#### Global Power Spectra in the Lβ and Hβ Sub-Bands, and δ Band

As shown in Figure 2C, both Lβ and Hβ GPS were significantly lower in patients [−4.7 (2.0) and −7.7 (1.9) dB, respectively] than in controls [−1.4 (2.9) and −5.4 (1.2) dB, respectively] (*p* = 0.004 for both EEG markers). There was a significant effect of frequency [*F*(2, 40) = 225, 93, *p* < 0.001] and of group [*F*(1, 20) = 12.37, *p* = 0.002] but no interaction [*F*(2, 40) = 2.41, *p* = 0.102]. On the contrary, there was no statistical difference in the δ GPS between patients [2.0 (1.7) dB] and healthy participants [3.4 (1.7) dB; *p* = 0.069] (Figure 2C). The significance threshold (α = 0.05) was corrected to α_*c*_0.017 with the Bonferroni method for multiple (*n* = 3) comparisons.

After normalizing Lβ and Hβ GPS to the GPS over the full range of frequencies band-filtered for data analysis (see section “Materials and Methods” for details), CNP patients still had significantly lower Lβ GPS compared with controls [respectively, 3.7 (1.4) and 5.7 (2.8) dB, *p* = 0.041], whereas the decrease noticed in the Hβ frequency range lost significance [0.8 (1.4) and 1.6 (1.1) dB, *p* = 0.123] (Figure 2D).

#### Power Maxima and Minima Localization

As an attempt to localize the maximal and the minimal β power in the sensor-space, we computed β GPS for the four defined quadrants (GPS_q_; see section “Materials and Methods” for the detailed procedure) and considered the quadrant holding the maximal GPS_q_ (GPS_qmax_) and the minimal GPS_q_ (GPS_qmin_) as the localization of, respectively, the β maximum and minimum. In the Lβ range, most patients (regardless of their predominant pain side) and controls displayed GPS_qmax_ in the posterior, left region (75 and 80%, respectively). The pattern of GPS_qmax_ localization was different in the Hβ range: 58.3% of patients showed the maximal averaged power in the anterior, left quadrant, whereas 70% of controls displayed their GPS_qmax_ within the right hemisphere (equally distributed in anterior and posterior regions). The GPS_qmin_ was variably distributed in controls (Lβ frequency: 70% in the anterior left, 17% in the posterior right; Hβ frequency: 40% in the anterior left and 50% in the posterior right), and in CNP patients [in the Lβ range: 50% in the anterior left quadrant, 33% in the anterior right, and 17% in the posterior right; in the Hβ range: 50% in the posterior right, the rest being equally observed (17%) in the three other quadrants], no relationship being observed with the predominant pain side. Here also, no remarkable difference was observed between patients and controls.

### Correlations Between Electroencephalographic Markers and Pain Intensity in Patients

The GPS was clearly lower in patients than in controls, both in the Lβ and in the Hβ frequency ranges. Thus, we expected a negative correlation between the VAS and the GPS for both EEG markers. On the day of the EEG recording, patients had pain levels at the limit of significance (VAS_d_ ∼3) (Mantha et al., 1993; Jensen et al., 2003; see Table 2). When relating their VAS_d_ to the GPS, no significant correlation was found, neither in the Lβ range (*R* = −0.453; *p* = 0.139) nor in the Hβ range (*R* = −0.349; *p* = 0.349). Interestingly, when we further discriminated between patients with nonsignificant pain (VAS_d_ < 3) and those with significant pain (VAS_d_ ≥ 3), no correlation was found in the absence of significant pain (*n* = 6) (Lβ: *R* = −0.418; *p* = 0.409; Hβ: *R* = 0.253; *p* = 0.629), whereas in patients with VAS_d_ ≥ 3 (*n* = 6), a strong correlation was now observed both in the Lβ (*R* = –.931; *p* = 0.007), while only at the edge of significance in the Hβ range (*R* = −0.805; *p* = 0.053). None of the two GABAergic markers was correlated to the VAS_2w_ (Lβ: *R* = −0.133; *p* = 0.680; Hβ: *R* = −0.281; *p* = 0.376), even when splitting between patients with VAS_2w_ < 3 and VAS_2w_ ≥ 3 (data not shown). The GPS in the δ range did not correlate neither with the VAS_d_ (*R* = −0.385; *p* = 0.216 for) nor with the VAS_2w_ (*R* = 0.282; *p* = 0.375), even when only considering patients with VAS ≥ 3 (data not shown).

Upon normalization, no correlation was anymore observed between Lβ GPS and VAS_d_ (*R* = −0.061, *p* = 0.851), even when solely evaluated in CNP patients with VAS_d_ ≥ 3 (*R* = −0.118, *p* = 0.824). There was no correlation between Hβ GPS and VAS_d_ (data not shown).

### Relation Between Mood and Pain

Because mood interacts with pain (Klyne et al., 2018), participants were administered the HAD questionnaire (see section “Materials and Methods”) (Zigmond and Snaith, 1983; Bjelland et al., 2002). Patients (*n* = 12) were moderately anxious [HAD_anx_ sub-score; 8.9 (3.6)] but not controls [5.3(2.8)] [*t*(20) = 2.541, *p* = 0.019], and the depression sub-score was normal in both groups [5.5 (4.3) and 2.2 (1.3), respectively]. Interestingly, the HAD_anx_ positively correlated with the VAS_d_ in patients (*R* = 0.755; *p* = 0.003) most probably due to the subgroup with VAS_d_ ≥ 3 (*n* = 6), which was the only one displaying a significant, positive correlation (*R* = 0.895; *p* = 0.016), contrary to the one with VAS_d_ < 3 (*R* = 0.375; *p* = 0.464). No correlation was found between VAS_2w_ (*n* = 12) and HAD_anx_ (data not shown). Altogether, these observations suggest that anxiety and pain intensity interacted daily but not on average. It was also interesting to assess the impact of pain on mood state. BPI question no. 9b (BPI_mood_) is specifically related to this issue. The BPI_mood_ score was highly correlated to HAD_anx_ (*R* = 0.711, *p* = 0.01). Thus, pain-diseased patients were anxious and had their pain intensity increasing with anxiety level, and the influence of pain on their mood was measurable in the anxiety domain. However, this study was not specifically designed to measure this interaction. It should be noted that no correlation was observed between the HAD_anx_ score and all studied EEG markers.

## Discussion

In the present pilot study, we recorded β EEG oscillations as an indicator of brain GABAergic activity (Christian et al., 2015) in patients with lower limb CNP of peripheral origin and matched controls, with the hypothesis that the β EEG activity could be a biomarker of CNP. More specifically, given the increased cortical excitation reported in chronic pain situations (see later discussion), we expected the (inhibitory) GABAergic signaling (and hence the β EEG activity) to decrease in patients.

In our analyses, we considered the GPS as a quantitative measurement of β EEG oscillatory activity. The most important result of our study is that, in accordance with our assumptions, the β GPS was significantly lower in patients than in healthy controls both in the Lβ and in the Hβ frequency sub-bands but not in the δ range (analyzed as a negative control). Our results are in accordance with transcranial magnetic stimulation, magnetic resonance spectroscopy, and EEG-based measurements performed in chronic pain patients, which found, respectively, decreased cortical inhibition (Barr et al., 2013), cortical GABA concentrations (Baumgarten et al., 2016), and lower γ oscillations (as GABAergic EEG marker) (Barr et al., 2013) in pain controlling regions. However, some studies reported rather increased β activity in neurogenic pain (Sarnthein et al., 2006). Beyond the heterogeneity of studied populations (i.e., mixed etiologies of neuropathic pain), several methodological issues can be raised: acquired data were down-sampled from 512 to 64 Hz, with a high risk of bias in the final output considering the frequency range of interest; data acquired with eyes closed were pooled together with those recorded in eye-open conditions, which is reported to dramatically modify both the topography and the magnitude of different frequency components (Barry et al., 2007). In addition, the wakefulness state was not segregated from sleepiness or drowsiness in these studies, with the great risk of slowing down the whole EEG recording pattern (Brown et al., 2012). Finally, the β frequency domain was segmented with different cutoffs. Other studies in patients with fibromyalgia, a disease in which mechanisms generating chronic pain are still elusive, reported at the same time increased (Lim et al., 2016), unchanged (Fallon et al., 2018), or decreased (Mhalla et al., 2010) cortical inhibition. Our study was conducted in a homogenous population (patients suffered all from neuropathic pain due to lesions in lower limb peripheral nerves), and data analysis was standardized using validated methods [selection of data recorded only in awaken state (Diaz et al., 2016) and β frequency segmentation (von Rotz et al., 2017)] in accordance with the two peaks observed in EEG data. Thus, the present results can be considered as reliable under the conditions in which the study was performed. The fact that normalization did not vanish the significant decrease adds up to the robustness of this finding. Furthermore, our data fit better with the current understanding of the pathophysiological mechanisms leading to chronic pain, i.e., a decreased brain inhibition underpinned by a deficiency in GABAergic signaling (Parker et al., 2016; Pelletier et al., 2017; Thibaut et al., 2017), which seems to be more specifically associated with neuropathic pain (Schwenkreis et al., 2010; Henderson et al., 2013; Parker et al., 2016).

The Lβ (but not the Hβ or δ) GPS was highly correlated with pain intensity when the latter was significant (Mantha et al., 1993; Collins et al., 1997), reinforcing our working hypothesis. However, the observed correlation disappeared after normalization, as well as significance of Hβ GPS differences between CNP patients and controls, which suggests that there could be a bias related to other differences between these two groups, such as in the GPS computed over the full range of frequencies. Alternatively, considering that there exists no standard among normalization methods that fits all EEG data (Bogaarts et al., 2016), one could not exclude the hypothesis that the discrepancy noticed between non-normalized and normalized data is only the effect of a nonsuitable normalization procedure. On the other hand, the small sample size could itself explain this lack of significant results after normalization. Overall, it seems that interpretation of differences between non-normalized and normalized data is possibly not unique and should be left open for further investigations in larger studies and an adequate normalization procedure clearly determined.

In conclusion, the association between Lβ GPS, supposedly measuring brain GABAergic activity, and pain intensity suggests that Lβ oscillations is a potential CNP biomarker, even if the measured modality of pain is still to be more precisely determined and these preliminary results confirmed in larger investigation settings.

Given the major contribution of GABA to pain regulation, we expected the reduction observed in the GABAergic markers of pain (reflected by the GPS_qmin_) to be located within the cerebral hemisphere contralateral to the pain side (Barr et al., 2013). To our great surprise, GPS_qmin_ localization was irrespective of patients’ pain side in the Lβ as in the Hβ frequency range. Furthermore, the distribution of both GPS_qmin_ and GPS_qmax_ in Lβ and Hβ seemed to be similar in patients and controls, with comparable lateralization trends in the two groups. This finding would suggest a relationship with nociception rather than with pathological pain. Interestingly, most patients and controls (all right-handed) had their Lβ GPS_qmax_ located in the left and posterior hemisphere. A recent study conducted in healthy right-handed individuals found a positive and linear correlation between the β EEG oscillatory activity and the endogenous concentration of GABA in the somatosensory cortex only in the left hemisphere (Baumgarten et al., 2016). Likewise, chronic pain diseased-patients displayed left-lateralized structural changes in the sensory-motor cortex areas (Baliki et al., 2012) and in the left posterior thalamus (Schmidt-Wilcke et al., 2007) (which has nociceptive projections (Kramer et al., 2017a,b) to the somatosensory cortex (Viaene et al., 2011; Castejon et al., 2016). Thus, it seems that the GABAergic neurotransmission involved in nociception (more specifically measured by Lβ GPS) is lateralized to the dominant handedness hemisphere of both healthy and chronic pain populations, in contradiction with previous data stating bilateral or right-sided pain processing inputs (Coghill et al., 2001; Favilla et al., 2014).

If we consider that Lβ GPS was strongly correlated to pain intensity, we could further assume that Lβ might represent the sensory modality of pain (supposedly measured by the VAS (Boggero and Carlson, 2015). On the other hand, the correlation between Hβ GPS and pain intensity (VAS_d_) was only at the edge of significance, whereas VAS_d_ was positively correlated with HAD_anx_. Interestingly, emerging data suggest that Hβ GPS could possibly be related to anxiety and similar mood disorders, pointing out rather an increase of β oscillations in this context (Roohi-Azizi et al., 2017; Ribas et al., 2018). In contrast, our data failed to show that the correlation between Hβ GPS and HAD_anx_. Since HB GPS_qmax_ was mostly located in the anterior quadrants, Hβ GPS could be related to the affective component of pain (Boggero and Carlson, 2015), of which controlling structures are located more anteriorly in the brain (Kuner and Kuner, 2020), thereby making the evoked link with the affective life. However, because the present study was not designed to distinguish between the sensory and the affective components of pain, further investigations are needed to explore more precisely the hypotheses mentioned earlier. Additionally, localization in the sensor space of EEG signals is subject to discussion (Rutkove, 2007), whereas leg representation in the sensory homunculus is close to the midline, making it difficult to reliably determine the lateralization side. More accurate analyses (e.g., source localization) are needed to get more insight into this issue.

Overall, our data suggest a plausible relation between CNP and the GABAergic EEG makers investigated and, more specifically, the Lβ GPS, in accordance with our working hypothesis. The Hβ GPS, less consistently associated with pain, could be related to affective parameters such as anxiety or the affective component of pain. However, we should be cautious in generalizing our conclusions or extrapolating them to other settings given the low number of participants in this study and the differences induced by data normalization. In addition, it is difficult to further specify, at this stage, what is exactly being measured through these EEG markers, knowing that β oscillations have also been associated with other brain functions or pathological situations (Mallet et al., 2008; Mirzaei et al., 2017; Clarke et al., 2018; Mane et al., 2018). On the other hand, one could argue that the found differences and correlations are related in general to sensory symptoms of the patients’ neuropathy and not specific to pain. However, the patients’ neurological examination found only minor sensory deficits unequally distributed among patients. If sensory deficits had played a significant role in these findings, the consistency of data would not be as remarkable as observed. Thus, we think that the data obtained are, on a somatic level, more likely to be related to pain.

## Conclusion and Future Directions

The present pilot study aimed at studying the β EEG oscillatory activity as a GABAergic marker indexing chronic pain, having in mind the need for a better and more objective evaluation and mechanistic classification of pain to improve analgesic treatment efficiency.

In line with our main hypothesis, we found that CNP patients had lower EEG Lβ GPS than healthy controls and that Lβ power was negatively correlated to pain intensity. However, when data were normalized to the total GPS, the correlation between Lβ GPS and pain intensity was not found anymore. It also seemed that Lβ-related nociceptive GABAergic neurotransmission could be lateralized in somatosensory brain areas of the handedness-dominant hemisphere, in contradiction with the current knowledge.

These preliminary results, although needing confirmation, open the possibility for an objective evaluation of pain by means of a short (24-min) EEG recording at rest. Furthermore, the marker we studied seems to be solely related to GABAergic signaling (and presumably to GABA cortical concentration). This should therefore open a way to a mechanism-based classification of pain and subsequently toward a more personalized GABA-based analgesic therapy depending on the extent to which the GABAergic signaling is involved.

In the future, it would be interesting to study the same markers in other pain models, assessing the role played by GABA in their underlying mechanisms.

## Data Availability Statement

The raw data supporting the conclusions of this article will be made available by the authors, without undue reservation, to any qualified researcher.

## Ethics Statement

The studies involving human participants were reviewed and approved by the Commission cantonale d’éthique et de la recherche sur l’être humain-Vaud (CER-VD). The patients/participants provided their written informed consent to participate in this study.

## Author Contributions

MT: major contribution to the patients’ recruitment, contribution to the controls’ recruitment, major contribution to the data collection, and manuscript writing and revision. CM: contribution to the study conception and initiation, major participation to the controls’ recruitment, major contribution to the data collection, data interpretation, and data analysis, and contribution to the manuscript writing and revision. CW: major contribution to the data analysis and data interpretation and contribution to the manuscript writing and revision. GM: major participation to the patients’ recruitment and study initiation and contribution to the data discussion. TK: contribution to the study conception and initiation, participation in the patients’ recruitment, manuscript revision, and language editing. DC: major contribution to the manuscript revision and language editing. MM: participation in the data collection, contribution to the data analysis, manuscript writing, and manuscript revision. J-MA: contribution to the data interpretation and significant contribution to the manuscript revision. JC: study conception and initiation, data collection, data analysis, main data interpretation, manuscript writing, and manuscript revision. All authors contributed to the article and approved the submitted version.

## Conflict of Interest

The authors declare that the research was conducted in the absence of any commercial or financial relationships that could be construed as a potential conflict of interest.

## Abbreviations

α: Alpha
BPI: Brief pain inventory
BPI_mood_: Brief pain inventory score related to the mood (question n ° 9b)
CNP: Chronic neuropathic pain
δ: Delta
dB: Decibel
DN4: Douleurs neuropathique 4 questions
EEG: Electroencephalogram/Electroencephalography/Electroencephalographic
γ: Gamma
GABA: Gamma-aminobutyric acid
GPS: Global power spectrum
GPS_q_: Global power spectrum of a scalp quadrant
GPS_qmax_: the highest power of the Global power spectrum in a scalp quadrant
GPS_qmin_: the lowest power of the global power spectrum in a scalp quadrant
HAD: Hospital anxiety and depression
HAD_anx_: Hospital anxiety and depression subscore for anxiety
Hβ: High beta
Hz: Hertz
Lβ: Low beta
θ: Theta
TMS: Transcranial magnetic stimulation
VAS: Visual analog scale
VAS_d_: Visual analog scale recorded the same day as the electroencephalogram
VAS_2w_: Visual analog scale of the last 2 weeks before electroencephalogram.
